# Sarcopenia and related musculoskeletal phenotypes in patients considered for spinal cord stimulation: a scoping review

**DOI:** 10.3389/fmed.2026.1867982

**Published:** 2026-07-16

**Authors:** Jakub Wiśniewski, Anna Barbara Marcinkowska, Mateusz Szczupak

**Affiliations:** 1 Department of Neurosurgery, Nicolaus Copernicus Hospital, Gdańsk, Poland; 2 Applied Cognitive Neuroscience Lab, Department of Neurophysiology, Neuropsychology and Neuroinformatics, Medical University of Gdańsk, Gdańsk, Poland; 3 2nd Department of Radiology, Medical University of Gdańsk, Gdańsk, Poland; 4 Department of Anaesthesiology and Intensive Therapy, Nicolaus Copernicus Hospital, Gdańsk, Poland

**Keywords:** body composition, body mass index, chronic neuropathic pain, frailty, obesity, psoas, sarcopenia, spinal cord stimulation

## Abstract

**Introduction:**

Musculoskeletal phenotype has not been systematically characterized in spinal cord stimulation research, although body habitus has occasionally been examined as a possible outcome modifier.

**Aim of study:**

To map the available evidence on sarcopenia, frailty, body composition, and related muscle-related variables in adults considered for or treated with spinal cord stimulation for chronic pain.

**Methods:**

This scoping review followed PRISMA-ScR guidance. PubMed/MEDLINE, Embase, and Scopus were searched from inception to 13 to 14 April 2026 without language restrictions. The search targeted spinal cord stimulation, chronic pain, and musculoskeletal phenotypes. Records were deduplicated by DOI, PMID, and normalized title. Title and abstract screening were performed independently by two reviewers. A formal risk-of-bias assessment was not conducted because the objective was evidence mapping rather than pooled-effect estimation. A descriptive appraisal of the study design and the definition of exposure was incorporated into the synthesis.

**Results:**

The searches identified 358 records. After removing 135 duplicate records, 223 records underwent title and abstract screening. Eleven full-text articles were assessed, and eight studies were included. No study diagnosed sarcopenia using consensus-based criteria. No study incorporated handgrip strength, gait speed, chair-stand testing, or dual-energy x-ray absorptiometry-derived lean mass — the standard tools recommended by current international consensus for sarcopenia diagnosis. The identified literature focused mainly on body mass index, obesity, and, in one study, psoas or iliopsoas-based imaging metrics. Two cohort studies reported less favorable outcomes with increasing body mass index, whereas two single-center series showed that severe or morbid obesity did not preclude technically successful treatment. One study suggested sex-specific associations between psoas-based measurements and selected pain or depression outcomes.

**Conclusion:**

Direct evidence on sarcopenia in spinal cord stimulation populations is absent. The current literature addresses body habitus more often than muscle phenotype and relies predominantly on body mass index, which cannot be used as a surrogate for sarcopenia. Future prospective cohorts should incorporate standardized measures of muscle strength, physical performance, and muscle quantity or quality.

## 1 Introduction

Sarcopenia is a progressive skeletal muscle disorder characterized by loss of muscle strength as its primary defining feature, confirmed by reduced muscle quantity or quality, and associated with impaired physical performance in its most severe form (1). First operationalized as a geriatric syndrome, sarcopenia is now recognized across a broad range of chronic conditions in which physical inactivity, systemic inflammation, and neuroendocrine dysregulation converge to accelerate muscle loss (1, 2). The clinical consequences extend beyond physical function: sarcopenia is independently associated with increased fall risk, prolonged hospitalization, greater post-surgical morbidity, and reduced health-related quality of life (1). Contemporary diagnostic frameworks, including the revised European Working Group on Sarcopenia in Older People consensus and the Global Leadership Initiative on Malnutrition criteria, have standardized assessment using handgrip dynamometry, chair-stand performance, gait speed, and imaging-derived measures of skeletal muscle index, enabling reproducible phenotyping across clinical settings (1).

The clinical relevance of sarcopenia has been demonstrated in several pain and surgical populations that share pathophysiological features with chronic neuropathic pain. In patients undergoing lumbar spine surgery, preoperative sarcopenia is associated with significantly worse functional recovery assessed by the Oswestry Disability Index, greater back and leg pain improvement deficits, longer hospitalization, and higher rates of discharge to rehabilitation facilities compared with non-sarcopenic patients (3, 4). In a prospective cohort of older adults with chronic low back pain, sarcopenia, defined by EWGSOP2 criteria, was present in nearly one-third of patients seeking physical therapy, and these patients reported significantly higher pain intensity and disability than those without sarcopenia (5). In patients with fibromyalgia, muscle strength and physical performance are significantly lower than in age-matched controls according to EWGSOP2-based assessment, with higher SARC-F scores indicating elevated sarcopenia risk even in younger women (6). Taken together, this evidence indicates that sarcopenia is a clinically actionable phenotype across chronic pain and surgical populations, and that the absence of equivalent data in spinal cord stimulation research represents a genuine evidence gap. Chronic pain and sarcopenia share a bidirectional relationship that is biologically plausible and clinically underappreciated. Persistent pain reduces physical activity, promoting disuse atrophy and accelerating the decline in muscle strength and mass. Opioid analgesics, commonly prescribed in refractory neuropathic pain, independently impair anabolic signaling and contribute to hypogonadism, further compounding muscle loss (7, 8). Systemic low-grade inflammation, a feature of both chronic neuropathic conditions and sarcopenia, drives catabolic activity through pro-inflammatory cytokines, including interleukin-6 and tumor necrosis factor-alpha (1). Body mass index does not distinguish adiposity from lean mass and cannot serve as a proxy for sarcopenia; a patient with sarcopenic obesity may carry a normal or elevated body mass index while having substantially compromised functional capacity.

Spinal cord stimulation is an established treatment option for selected patients with chronic refractory neuropathic pain, including failed back surgery syndrome, complex regional pain syndrome, and painful diabetic neuropathy. Treatment response remains heterogeneous, and existing predictor research has focused primarily on diagnosis, psychological factors, smoking, and device-related variables (9–12). Given that chronic pain populations are disproportionately affected by physical deconditioning, opioid exposure, and the systemic inflammatory burden that promotes sarcopenia, it is plausible that muscle phenotype influences treatment outcomes. Whether sarcopenia or related constructs have been studied in this context, and whether evidence exists to support their incorporation into candidate selection or outcome prediction, remains unknown. The present review was undertaken to map the available evidence on sarcopenia, frailty, body composition, and related musculoskeletal variables in adults considered for or treated with spinal cord stimulation for chronic pain. The aim was not to estimate a pooled treatment effect, but to define what has been studied directly, what has been evaluated only indirectly, and where the current evidence gap lies.

## 2 Materials and methods

This study was designed as a scoping review because the available literature was expected to be limited, heterogeneous, and methodologically diverse. The review was informed by current JBI scoping review guidance and reported in accordance with the PRISMA extension for Scoping Reviews (2, 13).

The review question was structured according to the Population, Concept, and Context framework. The population comprised adults with chronic pain who were considered for or treated with spinal cord stimulation. The concept included sarcopenia and related musculoskeletal phenotypes, including frailty, body composition, muscle mass, muscle quality, muscle strength, obesity, body mass index, and imaging-based muscle metrics. The context was defined as clinical outcomes after spinal cord stimulation, including pain relief, functional outcomes, revision, explantation, and procedure-related complications.

Eligible studies reported original human research, included adults aged 18 years or older, evaluated spinal cord stimulation for chronic pain, reported at least one clinically relevant outcome, and assessed at least one musculoskeletal or body composition variable. The 18-year lower age limit was applied deliberately, rather than restricting eligibility to older adults, for three reasons: the review aimed to map the full breadth of available evidence; the spinal cord stimulation candidate population is predominantly middle-aged rather than geriatric; and reduced muscle mass and function in chronic pain are not confined to older age, given the contributions of disuse, opioid exposure, and systemic low-grade inflammation. Imposing a higher age floor *a priori* would have excluded most of the relevant cohorts and pre-judged the evidence gap being characterized.

Reviews, editorials, conference abstracts without full text, animal studies, case reports, and case series with fewer than 10 participants were excluded. Studies that evaluated only post-treatment weight change or metabolic biomarkers without a baseline musculoskeletal phenotype were also excluded.

The literature search was performed in PubMed/MEDLINE on 13 April 2026 and in Embase and Scopus on 14 April 2026. No language restrictions were applied. Reference lists of included studies were screened manually. The search strategy was developed iteratively by the authors and refined through pilot testing of known relevant records. Formal input from a health sciences librarian was not available and is acknowledged as a limitation.

Records were combined across databases and deduplicated by DOI, PMID, and normalized title matching. Study selection proceeded in two stages: title and abstract screening, followed by full-text review. All 223 records remaining after deduplication underwent independent title and abstract screening by two reviewers; disagreements were resolved by consensus. Full-text review of eleven articles and data extraction were performed by one reviewer and verified by the second.

The PubMed search string was: ((“spinal cord stimulation”[Title/Abstract] OR “spinal cord stimulator”[Title/Abstract]) AND (pain[Title/Abstract] OR neuropath*[Title/Abstract] OR “persistent spinal pain syndrome”[Title/Abstract] OR “failed back surgery syndrome”[Title/Abstract] OR CRPS[Title/Abstract] OR “complex regional pain syndrome”[Title/Abstract])) AND (“sarcopenia”[Title/Abstract] OR “frailty”[Title/Abstract] OR “muscle mass”[Title/Abstract] OR “muscle strength”[Title/Abstract] OR “muscle quality”[Title/Abstract] OR “body composition”[Title/Abstract] OR “body mass index”[Title/Abstract] OR BMI[Title/Abstract] OR obesity[Title/Abstract] OR psoas[Title/Abstract] OR iliopsoas[Title/Abstract]). Equivalent syntax adapted to Embase and Scopus is provided in Supplementary Appendix S1.

Data extraction included study design, setting, sample size, spinal cord stimulation indication, exposure variable, outcome measures, and findings relevant to the review question. A formal risk-of-bias assessment was not undertaken because the objective of the review was to map the extent and characteristics of the evidence rather than to support pooled inference. To address methodological concerns, the synthesis includes a descriptive appraisal of study design, exposure definition, and outcome heterogeneity.

## 3 Results

The three database searches yielded 358 records: 64 from PubMed/MEDLINE, 234 from Embase, and 60 from Scopus. After removing 135 duplicate records, 223 records underwent title and abstract screening. Eleven full-text articles were assessed for eligibility, three were excluded at full-text review, and eight studies were included in the final synthesis.

All included studies had an observational design. Six studies evaluated clinical cohorts treated with spinal cord stimulation, one study used a national inpatient administrative database, and one multicenter study examined infection risk after implantation or revision. No included study diagnosed sarcopenia using consensus-based criteria. No study used handgrip strength, chair-stand testing, gait speed, short physical performance battery, or dual-energy x-ray absorptiometry-derived muscle mass.

Five studies addressed body composition or body habitus as a baseline characteristic (9–11, 14, 15). Four of these focused on body mass index or morbid obesity, whereas one evaluated psoas or iliopsoas-based imaging metrics as a proxy for muscle phenotype (16). Three further studies mapped adjacent evidence relevant to the review question, including obesity as a predictor in a model, obesity and infection risk, and inpatient obesity trends among patients with spinal cord stimulator implants (9, 12, 17).

De La Cruz et al. analyzed 57 patients with six-month follow-up and found that body mass index did not affect global outcome ratings in that cohort, whereas smoking and drug use showed stronger signals of failure (9). Marola et al. compared patients above and below a body mass index threshold of 36.5 and found less improvement in Beck Depression Inventory scores at 6 months and 1 year, and less improvement in pain catastrophizing at 1 year among patients with higher body mass index (10).

Mekhail et al. studied 181 patients with chronic spine-related pain and reported a statistically significant negative association between higher body mass index and the effectiveness of spinal cord stimulation at 6 and 12 months. Their multivariable analysis suggested an approximately 2% reduction in efficacy for every one-unit increase in body mass index, whereas opioid utilization did not change significantly across body mass index strata (11).

Sommer et al. examined 73 paddle lead trials and found that severe obesity did not preclude procedural success. Overall trial success was 82.2%, and success was higher in the body mass index 40 or greater cohort than in patients with a lower body mass index (14). Bharthi et al. reported outcomes in 67 morbidly obese patients with a mean preoperative body mass index of 44.47 kg/m^2^ and found no neurological complications, 3 culture-positive infections, 9 superficial wound dehiscence events, and postoperative improvement in pain scores (15).

Hoelzer et al. evaluated 2,737 unique implants or revisions and found an overall infection rate of 2.45%. Obesity did not independently increase the risk of infection in that analysis (12). Orhurhu et al. identified 3,893 hospitalized patients with spinal cord stimulator implants and showed that obesity diagnoses became more frequent over time, although no significant difference in in-hospital cost was observed after matching (17).

Sheldon et al. provided the only study to move beyond body mass index and to assess a direct muscle-related imaging proxy. In 73 patients with baseline and one-year outcome data, iliopsoas cross-sectional area and derived ratios were associated with selected pain and depression outcomes in a sex-specific manner. These findings represent the closest available approximation to muscle phenotyping in the identified literature on spinal cord stimulation, but they do not constitute a consensus-defined assessment of sarcopenia (Table 1) (16).

**Table 1:** Evidence mapping of included studies.

Study	Design	Sample	Exposure	Main finding	Age, y (mean ± SD; range)
De La Cruz et al. (2015) (9)	Retrospective review of prospective database	57 patients with 6-month follow-up	BMI among several predictors	BMI did not affect 6-month outcome; smoking showed stronger association with failure	NR (47.8/51.2 by outcome)
Marola et al. (2017) (10)	Retrospective analysis of prospectively collected outcomes	77 patients	BMI ≥ 36.5 vs. < 36.5	Higher BMI associated with less BDI improvement and less PCS improvement in selected domains	52.1/52.3; 21–80
Hoelzer et al. (2017) (12)	Multicenter retrospective study	2,737 implants or revisions	Obesity as infection risk factor	Overall infection rate 2.45%; obesity not independently associated with infection	55.7 ± 14.5
Mekhail et al. (2019) (11)	Retrospective cohort	181 patients	BMI in 4 cohorts	Increasing BMI associated with lower effectiveness at 6 and 12 months	55 ± 12
Orhurhu et al. (2020) (17)	National inpatient sample analysis	3,893 hospitalized patients	Obesity diagnosis	Obesity more frequent over time; no significant matched difference in cost	56.2 ± 14.5
Sheldon et al. (2022) (16)	Retrospective MRI-based cohort	73 patients	Psoas/iliopsoas metrics	Sex-specific associations with selected pain and depression outcomes	56.3 ± 12.8; 28–81
Sommer et al. (2023) (14)	Retrospective cohort	73 paddle lead trials	BMI ≥ 40 vs. < 40	Severe obesity did not preclude trial success; higher success in BMI ≥ 40 cohort	58.1–62.6; pooled ≈60.7
Bharthi et al. (2023) (15)	Retrospective single-surgeon cohort	67 morbidly obese patients	Morbid obesity	No neurological complications; low infection rate; pain improvement observed	58.9 ± 11.4; 32–84

## 4 Discussion

This scoping review shows that spinal cord stimulation research has not yet operationalized sarcopenia as a formal baseline phenotype. None of the included studies used consensus-based diagnostic criteria and none incorporated standard measures such as grip strength, chair-stand performance, gait speed, or dual-energy x-ray absorptiometry-derived lean mass. The evidence base instead consists of studies that evaluate body mass index, obesity, or, in one case, psoas-related imaging metrics.

Why body mass index cannot substitute for sarcopenia assessment. The distinction between body mass index and sarcopenia is central to interpreting this literature. Body mass index does not distinguish adiposity from lean mass and cannot identify reduced muscle reserve, even when body weight is normal or elevated. The current body mass index literature, therefore, indicates that body habitus has been explored in spinal cord stimulation cohorts, but it does not show that sarcopenia has been evaluated directly (1, 10, 11).

The relationship between obesity and spinal cord stimulation outcome was not uniform across studies. Marola et al. and Mekhail et al. reported less favorable outcomes with increasing body mass index in selected domains (10, 11). By contrast, Sommer et al. and Bharthi et al. found that severe or morbid obesity did not preclude technically successful treatment and was associated with acceptable short-term safety profiles in their respective single-center series (14, 15). These findings suggest that obesity may influence some aspects of treatment response, but they do not support a simple view that obesity alone defines poor candidacy for spinal cord stimulation.

Only one study approached the topic from a muscle-centered perspective. Sheldon et al. examined iliopsoas-based measurements and identified sex-specific associations with selected pain and depression outcomes (16). These data are preliminary, but they demonstrate that muscle-related phenotyping is technically feasible in spinal cord stimulation cohorts and may reveal signals that are not captured by body mass index alone. At the same time, psoas size is an imaging proxy rather than a validated framework for sarcopenia.

**Figure 1:**
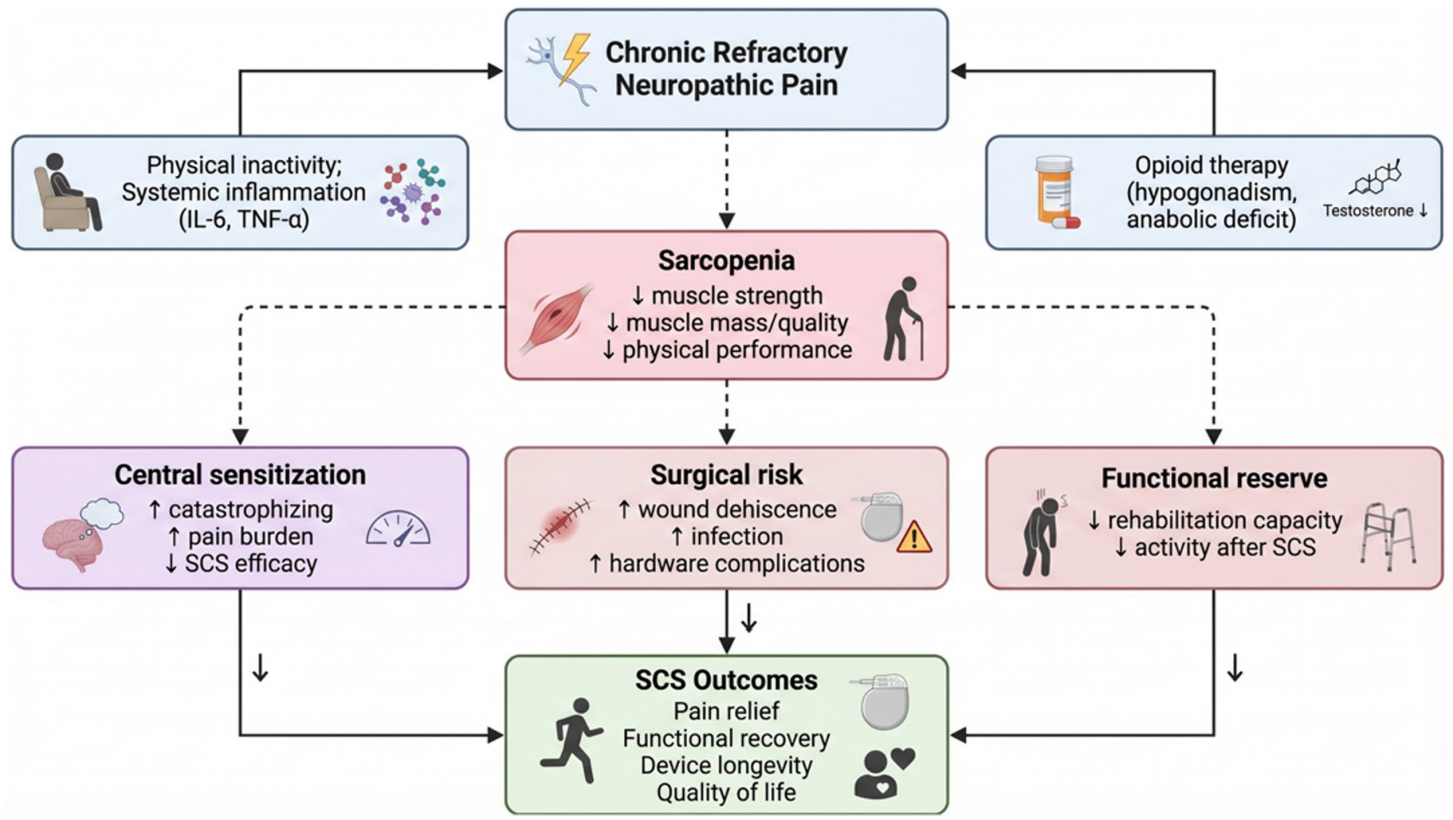
Proposed mechanistic pathways linking sarcopenia to spinal cord stimulation outcomes. Chronic refractory neuropathic pain promotes sarcopenia through two converging pathways: disuse-related skeletal muscle atrophy driven by physical inactivity and pain-related movement avoidance, and opioid-induced hypogonadism with impaired anabolic signaling. Systemic low-grade inflammation, characterized by elevated interleukin-6 and tumor necrosis factor-alpha, is a feature common to both chronic neuropathic conditions and sarcopenia and amplifies catabolic muscle loss through both pathways. The resulting sarcopenic phenotype (reduced muscle strength, low muscle mass or quality, and impaired physical performance) may influence spinal cord stimulation outcomes through three partially independent mechanisms. First, reduced muscle strength and impaired physical performance are associated with central sensitisation and heightened pain catastrophizing in chronic pain populations, both of which are established predictors of poorer neuromodulation response. Second sarcopenia increases peri-operative risk, including wound healing delay, superficial infection, and prolonged immobilization, each of which carries direct clinical relevance in the context of implantable device surgery. Third, low functional reserve limits post-implant rehabilitation capacity and physical activity engagement, potentially attenuating the functional gains achievable with effective pain relief. These pathways converge on the clinical outcomes most relevant to spinal cord stimulation: degree of pain relief, functional recovery, health-related quality of life, and device longevity. Arrows indicate hypothesized directional relationships derived from mechanistic and observational evidence in chronic pain and surgical populations; none has been directly tested in spinal cord stimulation cohorts. Dashed arrows indicate pathways supported by indirect or analogical evidence only. IL-6, interleukin-6; TNF-*α*, tumor necrosis factor-alpha; SCS, spinal cord stimulation. Created with BioRender.com.

Several biological mechanisms support the hypothesis that sarcopenia could influence spinal cord stimulation outcomes. In chronic pain populations—though not yet in spinal cord stimulation cohorts specifically—reduced muscle strength and impaired physical performance are associated with central sensitization and heightened pain catastrophizing, both of which are recognized predictors of poorer neuromodulation response (16, 17). Low skeletal muscle mass is associated with systemic inflammation and elevated circulating cytokines, which may modulate spinal excitability and reduce analgesic efficacy at the dorsal horn level. Sarcopenic patients are also at greater risk of postoperative complications, including wound healing delay, infection, and prolonged immobilization, all of which are clinically relevant in the context of implantable device surgery. Finally, frailty—which encompasses sarcopenia alongside nutritional deficiency and fatigue—independently predicts adverse outcomes after spine surgery and is increasingly evaluated in other implantable device populations such as cardiac resynchronization therapy and deep brain stimulation. The biological plausibility of sarcopenia as a modifier of spinal cord stimulation outcome is therefore substantial, even in the current absence of direct evidence (Figure 1). Clinical implications. Several clinical scenarios within the spinal cord stimulation workflow may be particularly susceptible to the influence of muscle phenotype. Patients with complex regional pain syndrome and prolonged limb immobilization represent a high-risk group for accelerated regional and systemic muscle loss, and their functional reserve at the time of implant may directly affect rehabilitation trajectory and treatment durability. Patients with opioid-induced hypogonadism—a recognized consequence of long-term opioid therapy in refractory chronic pain—face compounded anabolic deficiency that accelerates sarcopenia independently of age and physical activity. Patients with failed back surgery syndrome who have undergone multiple spinal procedures may present with substantial paraspinal muscle atrophy, fatty infiltration, and denervation changes that are not captured by body mass index but that may influence both the biomechanical stability of implanted hardware and the neurophysiological substrate for stimulation. In these subgroups, even a brief functional screen—handgrip dynamometry or a five-times chair-stand test—could stratify patients by functional reserve and inform peri-implant rehabilitation planning. Incorporating such assessments into routine pre-implant evaluation would not require major workflow restructuring at most neuromodulation centers and would generate the longitudinal data necessary to determine whether sarcopenia has prognostic value in this population. The absence of sarcopenia-specific studies may have several explanations. Historical research on spinal cord stimulation has emphasized psychological predictors, diagnosis-specific outcomes, device-related complications, and procedural factors. Body mass index is easily available in retrospective data and may therefore have served as a pragmatic but inadequate substitute for more informative musculoskeletal measures. Standard sarcopenia tools, such as handgrip dynamometry, chair-stand testing, the short physical performance battery, gait speed, and dual-energy x-ray absorptiometry, are not routinely incorporated into neuromodulation workflows. Even when computed tomography or magnetic resonance imaging is available, skeletal muscle metrics are rarely extracted in outcome research.

The included studies were predominantly retrospective, and several were single-center cohorts. Exposure definitions varied from dichotomized body mass index to morbid-obesity cohorts, infection risk factors, administrative obesity diagnoses, and psoas-derived imaging ratios. Outcome measures were similarly heterogeneous and included pain scores, psychosocial instruments, trial success, infection, hospital utilization, and wound complications. For this reason, a formal pooled synthesis would not have been appropriate.

The current evidence does not justify incorporating sarcopenia into routine spinal cord stimulation selection algorithms as an evidence-based predictor. It does, however, support a focused research agenda. Future prospective cohorts should incorporate at least one measure of muscle strength, such as handgrip dynamometry with established EWGSOP2 cut-offs (below 27 kg in men and below 16 kg in women indicating low strength); one measure of physical performance, such as gait speed below 0.8 m/s, a five-times chair-stand time above 15 s, or a short physical performance battery score of 8 or below indicating impaired performance; and one measure of muscle quantity or quality, such as dual-energy x-ray absorptiometry-derived appendicular lean mass index below 7.0 kg/m^2^ in men and below 5.5 kg/m^2^ in women, or computed tomography or magnetic resonance imaging-based skeletal muscle index at the L3 vertebral level using sex-specific thresholds. Applying these criteria would enable classification of probable sarcopenia (low strength alone) and confirmed sarcopenia (low strength plus low muscle quantity or quality), consistent with the EWGSOP2 algorithm. Where preoperative spinal imaging is already part of clinical care, standardized extraction of psoas cross-sectional area or psoas muscle index may offer a pragmatic, cost-neutral starting point for retrospective database studies. It should be noted, however, that psoas-based metrics carry specific limitations in chronic pain and spine surgery populations: psoas asymmetry due to scoliosis or unilateral pathology, degenerative fatty infiltration, and variability in axial slice selection can reduce the reproducibility and validity of these measures; interpretation should therefore be made alongside other muscle phenotype data where possible. Future studies should also specify the analytical role of sarcopenia explicitly in their design. Treated as a baseline characteristic, sarcopenia describes the functional status of the implant population. Treated as a moderator, it tests whether the effect of spinal cord stimulation on pain or disability differs between patients with and without impaired muscle phenotype. Treated as a mediator, it examines whether changes in physical function after implantation partially explain treatment-related improvements in quality of life. Each of these roles requires different methodological approaches and sample size calculations, and their conflation in future work should be avoided. Minimum design requirements for informative studies in this area include prospective recruitment with pre-implant assessment, standardized outcome measurement at 6 and 12 months, and *a priori* specification of the sarcopenia variable as a primary or secondary outcome. Multi-center registry designs, embedded within existing neuromodulation quality databases, would provide sufficient sample sizes to examine subgroup effects by indication, sex, and age, and would allow harmonization of measurement protocols across centers. Researchers should consider operationalizing sarcopenic obesity separately from sarcopenia and from obesity alone, given that the combination carries distinct prognostic implications and cannot be identified by body mass index. Frailty instruments, such as the Clinical Frailty Scale or the FRAIL questionnaire, are brief enough to be administered during outpatient screening visits and have demonstrated predictive validity in surgical populations; their feasibility in neuromodulation settings warrants prospective evaluation. Patient-reported functional outcomes—including physical function subscales of the PROMIS battery, the EQ-5D mobility domain, and the Oswestry Disability Index physical performance items—could serve as proxies for functional capacity in cohorts where formal sarcopenia testing is not available. Finally, mechanistic studies are needed to clarify whether sarcopenia modifies spinal cord stimulation outcomes through effects on pain sensitization, procedural risk, treatment adherence, or physical rehabilitation capacity. Translational work linking skeletal muscle mass to dorsal horn excitability and descending pain modulation would strengthen the biological rationale for future clinical trials. A collaborative registry embedded within existing neuromodulation databases would provide sufficient sample sizes to address these questions across multiple centers and indicators.

## 5 Limitations

The review has several limitations. First, although the search was expanded to three major databases and no language restrictions were applied, grey literature searching remained limited, and conference abstracts from neuromodulation meetings were not systematically searched. Second, formal input from a health sciences librarian was not available during strategy development. Third, no formal risk-of-bias assessment was undertaken because the objective was evidence mapping rather than pooled inference; to address this, the revised synthesis includes an explicit descriptive appraisal of study design and exposure heterogeneity. Fourth, the included evidence was restricted to studies that reported at least one musculoskeletal or body composition variable, potentially excluding cohorts in which such data were collected but not reported as a primary outcome. Fifth, and perhaps most fundamentally, the complete absence of prospective studies with pre-specified assessments of sarcopenia in the identified literature means that the review maps an evidence gap rather than synthesizing available data. This is not a limitation of the review methodology but a characteristic of the field; it underscores the need for prospectively designed cohorts and validates the mapping objective of the present work. Sixth, although a scoping review does not formally appraise publication bias, the small relevant literature is concentrated in studies framed around obesity and surgical complications, and null or unfavorable musculoskeletal findings may be under-reported; this directional reporting tendency constrains the completeness of the evidence map. Seventh, because the included cohorts were predominantly middle-aged and did not assess sarcopenia directly, the applicability of the synthesized evidence to older adults living with, or at risk of, sarcopenia remains uncertain and is an explicit priority for future age-stratified study.

The included evidence was also limited in scope. Most studies assessed body mass index or obesity rather than direct muscle phenotype, and only one study evaluated an imaging-based muscle proxy. The resulting evidence gap is therefore genuine, but the boundary between absent evidence and indirect evidence needs careful handling.

## 6 Conclusion

Direct evidence on sarcopenia in spinal cord stimulation populations is currently absent. The identified literature addresses body habitus more often than muscle phenotype and relies mainly on body mass index, which cannot serve as a surrogate for sarcopenia. A small number of studies suggest that obesity may influence some clinical outcomes, while one study indicates that psoas-based measurements may carry additional information. Future spinal cord stimulation research should incorporate standardized measures of muscle strength, physical performance, and muscle quantity or quality if the field is to determine whether sarcopenia has independent relevance to treatment outcome.

## Data Availability

The original contributions presented in the study are included in the article/Supplementary material, further inquiries can be directed to the corresponding author.
