# Supplementary material for: Sarcopenia and related musculoskeletal phenotypes in patients considered for spinal cord stimulation: a scoping review
Source: Front Med (Lausanne). 2026 Jul 16;13:1867982. doi: 10.3389/fmed.2026.1867982 (PMC13421899; doi:10.3389/fmed.2026.1867982)
Supplement: Supplementary file 1 [file Table_1.DOCX]

**Identification of studies via databases and registers**

**Records removed before screening:**

- Duplicate records removed (n=135)
- Records marked as ineligible by automation tools (n=0)
- Records removed for other reasons (n=0)

**Records identified from:**

- Databases (n=358)
- Registers (n=0)

**Identification**

**Records excluded:**

(n=212)

**Records screened:**

(n=223)

**Reports not retrieved:**

(n=0)

**Reports sought for retrieval:**

(n=11)

**Screening**

**Reports excluded:**

- Post-treatment weight or metabolic outcomes only (n=2)
- No baseline musculoskeletal phenotype variable (n=1)
- Other (n=0)

**Reports assessed for eligibility:**

(n=11)

**Studies included in the review:**

(n=8)

**Reports of included studies:**

(n=8)

**Included**
