# Supplementary material for: Sarcopenia and related musculoskeletal phenotypes in patients considered for spinal cord stimulation: a scoping review
Source: Front Med (Lausanne). 2026 Jul 16;13:1867982. doi: 10.3389/fmed.2026.1867982 (PMC13421899; doi:10.3389/fmed.2026.1867982)
Supplement: Supplementary file 2 [file Table_2.DOCX]

Supplementary Material

**Manuscript title: Sarcopenia and Related Musculoskeletal Phenotypes in Patients Considered for Spinal Cord Stimulation: a Scoping Review**

# Supplementary Appendix S1. Full search strategies

PubMed/MEDLINE, final search date: 13 April 2026

(("spinal cord stimulation"[Title/Abstract] OR "spinal cord stimulator"[Title/Abstract]) AND (pain[Title/Abstract] OR neuropath*[Title/Abstract] OR "persistent spinal pain syndrome"[Title/Abstract] OR "failed back surgery syndrome"[Title/Abstract] OR CRPS[Title/Abstract] OR "complex regional pain syndrome"[Title/Abstract])) AND ("sarcopenia"[Title/Abstract] OR "frailty"[Title/Abstract] OR "muscle mass"[Title/Abstract] OR "muscle strength"[Title/Abstract] OR "muscle quality"[Title/Abstract] OR "body composition"[Title/Abstract] OR "body mass index"[Title/Abstract] OR BMI[Title/Abstract] OR obesity[Title/Abstract] OR psoas[Title/Abstract] OR iliopsoas[Title/Abstract])

Records identified: 64

Embase, final search date: 14 April 2026

(('spinal cord stimulation'/exp OR 'spinal cord stimulation':ti,ab OR 'spinal cord stimulator':ti,ab) AND ('chronic pain'/exp OR 'neuropathic pain'/exp OR pain:ti,ab OR neuropath*:ti,ab OR 'persistent spinal pain syndrome':ti,ab OR 'failed back surgery syndrome':ti,ab OR crps:ti,ab OR 'complex regional pain syndrome':ti,ab) AND ('sarcopenia'/exp OR 'frailty'/exp OR 'body composition'/exp OR obesity/exp OR sarcopenia:ti,ab OR frailty:ti,ab OR 'muscle mass':ti,ab OR 'muscle strength':ti,ab OR 'muscle quality':ti,ab OR 'body composition':ti,ab OR 'body mass index':ti,ab OR bmi:ti,ab OR obesity:ti,ab OR psoas:ti,ab OR iliopsoas:ti,ab))

Records identified: 234

Scopus, final search date: 14 April 2026

TITLE-ABS(("spinal cord stimulation" OR "spinal cord stimulator") AND (pain OR neuropath* OR "persistent spinal pain syndrome" OR "failed back surgery syndrome" OR CRPS OR "complex regional pain syndrome") AND (sarcopenia OR frailty OR "muscle mass" OR "muscle strength" OR "muscle quality" OR "body composition" OR "body mass index" OR BMI OR obesity OR psoas OR iliopsoas))

Records identified: 60

# Supplementary Appendix S2. Data extraction form

The following variables were extracted from each included study:

- Author and year
- Country or setting
- Study design
- Sample size
- Spinal cord stimulation indication
- Musculoskeletal or body composition exposure variable
- Outcome measures
- Key findings relevant to the review question
- Notes on methodological limitations

# Supplementary Appendix S3. Full-text exclusions after eligibility assessment

| Reason | Count |
| --- | --- |
| Post-treatment weight or metabolic outcomes without baseline musculoskeletal phenotype | 2 |
| No baseline musculoskeletal phenotype variable relevant to the review question | 1 |

**
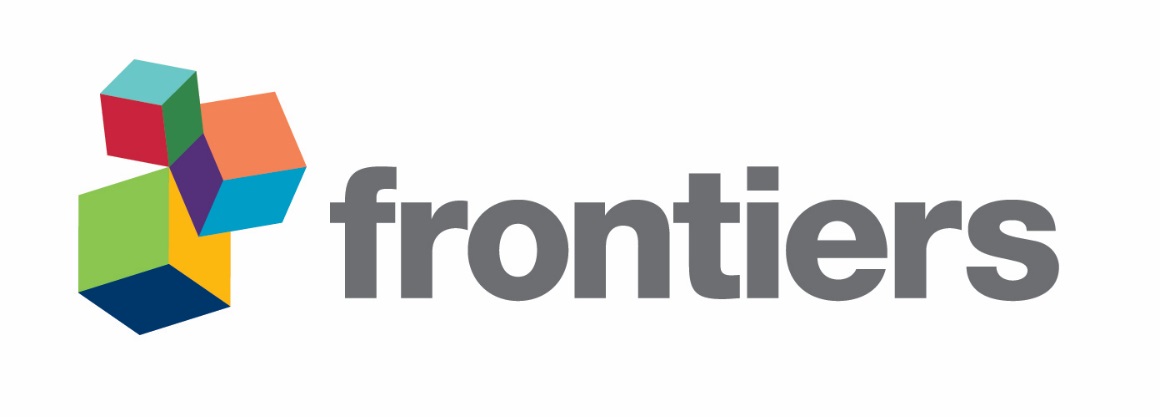
**
